# Supplementary material for: Epistasis between mutator alleles contributes to germline mutation spectrum variability in laboratory mice
Source: eLife. 2024 Feb 21;12:RP89096. doi: 10.7554/eLife.89096 (PMC10942616; doi:10.7554/eLife.89096)
Supplement: Supplementary file 1. [file elife-89096-supp1.docx]

| Gene name | Tissue name | # BXDs with expression data | Top significant marker | -log10(p) at top significant marker (GEMMA) | Additive effect of D allele on expression (GEMMA) |
| --- | --- | --- | --- | --- | --- |
| Ogg1 | Kidney | 53 | rsm10000004188 | 12.89 | -0.180 |
| Ogg1 | Liver | 50 | rsm10000004188 | 13.57 | -0.155 |
| Ogg1 | Spleen | 79 | rsm10000003418 | 4.73 | -0.056 |
| Ogg1 | Gastrointestinal | 46 | rs4173870 | 5.43 | -0.048 |
| Fancd2 | Gastrointestinal | 46 | rsm10000004199 | 8.60 | 0.133 |
| Ogg1 | Hippocampus | 67 | rsm10000004188 | 16.50 | -0.165 |
| Rad18 | Hippocampus | 67 | rsm10000003463 | 6.32 | 0.068 |
| Setmar | Hippocampus | 67 | rs13478947 | 11.03 | 0.141 |
| Mbd4 | Spleen | 79 | rsm10000004199 | 6.05 | 0.071 |

Supplementary *f*ile 1*.* Significant cis-eQTLs for DNA repair genes in various tissues identified using GeneNetwork.
